# Actively tunable THz filter based on an electromagnetically induced transparency analog hybridized with a MEMS metamaterial

**DOI:** 10.1038/s41598-020-77922-1

**Published:** 2020-11-30

**Authors:** Ying Huang, Kenta Nakamura, Yuma Takida, Hiroaki Minamide, Kazuhiro Hane, Yoshiaki Kanamori

**Affiliations:** 1 Department of Robotics, Tohoku University, Sendai, 980-8579 Japan; 2 Department of Finemechanics, Tohoku University, Sendai, 980-8579 Japan; 3 RIKEN Center for Advanced Photonics, RIKEN, Sendai, 980-0845 Japan

**Keywords:** Engineering, Electrical and electronic engineering, Mechanical engineering

## Abstract

Electromagnetically induced transparency (EIT) analogs in classical oscillator systems have been investigated due to their potential in optical applications such as nonlinear devices and the slow-light field. Metamaterials are good candidates that utilize EIT-like effects to regulate optical light. Here, an actively reconfigurable EIT metamaterial for controlling THz waves, which consists of a movable bar and a fixed wire pair, is numerically and experimentally proposed. By changing the distance between the bar and wire pair through microelectromechanical system (MEMS) technology, the metamaterial can controllably regulate the EIT behavior to manipulate the waves around 1.832 THz, serving as a dynamic filter. A high transmittance modulation rate of 38.8% is obtained by applying a drive voltage to the MEMS actuator. The dispersion properties and polarization of the metamaterial are also investigated. Since this filter is readily miniaturized and integrated by taking advantage of MEMS, it is expected to significantly promote the development of THz-related practical applications such as THz biological detection and THz communications.

## Introduction

The electromagnetically induced transparency (EIT) effect, resulting from quantum interference, has been extensively researched over the last few decades due to its elimination of absorption in a narrow window within a broad absorption region and its ability to achieve dramatic changes in dispersive properties^1–3^. This phenomenon was later mimicked in nonquantum systems by coupling harmonic oscillators. Specifically, the interference arising from the surface plasmon polariton can be generated by coupling the bright mode, which is directly coupled with the incident electric field, and the dark mode, which is excited by the bright mode^4–6^. Due to the mimicked similarity of the extremely changeable optical response and the rapid change in dispersion in a narrow region, the EIT plasmonic analogy in classical optical systems has garnered increasing attention yearly and can be widely used in the nonlinear^7^, sensor^8,9^, and slow-light^10^ fields. Metamaterials, as artificial optical materials, are a remarkable scheme for realizing a desired EIT plasmonic behavior through the design of a subwavelength structure; their potential has been demonstrated in various applications ranging from antennas^11^ to absorbers^12^, optical switches^13^, and optical buffers^14^ by generating an EIT-like effect. In 2014, Hokari et al*.*^15^ investigated the different EIT-like phenomena among silver, gold, and aluminum metamaterials consisting of a single wire and a wire pair at visible wavelengths and studied the EIT-like characteristics as functions of the constructional materials as well as the gap distance of the wires. In the same year, this group fabricated plasmonic metamaterials, achieving strong and sharp EIT-like characteristics at wavelengths around 820 nm with a transmittance of 72%^16^. In 2015, Nakamura et al*.*^17^ successfully demonstrated the EIT-like effect with gold metamaterials comprising a cut wire and a wire pair corresponding to the bright mode and dark mode, respectively.

In recent years, actively controllable EIT metamaterials have been considerably attractive due to the expected realization of dynamic modulation of the transmittance and dispersive characteristics, which is critical for optical practical applications^18–20^. Via several electron injection tuning approaches, such as liquid crystal control, use of the phase transition effect, and use of graphene resonators, regulation of the EIT analog can be achieved due to the tunable modification of optical constants of the medium^21–23^. However, some difficulties exist in the realistic fabrication of these schemes due to the challenges in integrating the tuning triggers for the system. On the other hand, the EIT-like behavior in metamaterials can be manipulated by breaking the symmetry of the structure or by varying the structure shape, namely, by regulating the coupling efficiency between the bright and dark resonators, which could be easily managed by microelectromechanical systems (MEMS) in advantageous ways. Moreover, only a small actuation force is required for the MEMS actuation system to work with a high response speed and low power consumption, which allows mechanically reconfigurable metamaterials to be competitive candidates for achieving active EIT-like behavior control^24^. Numerous studies have been conducted to modulate characteristics such as the transmission and spectral dispersion from the visible to near-infrared region based on MEMS metamaterials^25–29^. Liu et al*.*^30^ experimentally provided an EIT-based metamaterial working in the infrared region, the spectral response of which was obviously modified by inducing structure asymmetry driven by a MEMS actuation system, in which the transmittance for waves at 173 THz changed from below 30% to above 75%.

Recently, with the ever-increasing attraction of terahertz (THz) waves, which have great potential in communications, imaging, and sensors due to their outstanding performances, such as strong penetration and high resolution^31–34^, metamaterial-based devices that can control THz waves are being frequently reported^35–44^. In particular, dynamic regulation of THz waves is highly desired. Therefore, an active filter for manipulating the spectral response of THz waves is very attractive. Several studies utilizing reconfigurable metamaterials to modify the linewidth of transparency windows in the THz regime based on the EIT-like effect have been proposed^45–47^. However, for some applications, such as biodetectors and spatial communications, selecting a fixed transparency window in the THz region and altering its transmission properties may be more desirable. Since EIT metamaterials require a trade-off between a small loss and a large dispersion, the design of the reconfigurable EIT structure should carefully strike a balance^48^. In 2012, Gu et al*.*^49^ first demonstrated active control of an EIT analog at THz frequencies by using the optically tunable conductivity of the Si islands of a metamaterial on a sapphire substrate, which achieved controllable transmission from 85 to 50% for 0.74 THz waves, although the high cost and intractability of the sapphire substrate limit the commercialization and miniaturization of the metamaterial. In 2016, Prakash et al*.*^50^ experimentally proposed an out-of-plane MEMS-driven metamaterial for modulating THz waves based on the EIT-like effect, for which the transparency peak at 0.55 THz with a transmittance of 70% controllably dropped while the resonant frequency suffered a shift of 74 GHz as the structure changed. In 2020, Xu et al*.*^51^ numerically proposed a reconfigurable metamaterial on a silicon substrate to actively filter the waves at 0.65 THz by using an EIT analog. However, all previously reported MEMS metamaterial devices have substrates, and a difficult problem with a substrate is that it creates extra background intensity and interference waveforms, which adversely affect the optical properties of the device. In addition, few works have experimentally demonstrated EIT metamaterials incorporating MEMS to tune the transmittance for particular THz waves.

In this article, an active MEMS-driven metamaterial for manipulating THz waves at a consistent resonant frequency has been experimentally realized based on the EIT-like effect. The proposed metamaterial consists of a movable bar that functions as a bright-mode resonator and a fixed wire pair that acts as a dark-mode resonator. As the bar is displaced close to the wire pair through an electrostatic force by applying a drive voltage to the MEMS actuator, the transmittance of THz waves at the resonant frequency is controllably changed. The transmission phase shift associated with the dispersion properties and the polarization of the filter is also investigated. Moreover, the nature of the EIT analog in our metamaterial is analyzed by using numerical calculations and discussed. One of the most important features of this device is the lack of a substrate under the metamaterial. Conventionally, in MEMS metamaterials, the nonplanar bar-pair component that consists of a self-supporting movable part and a substrate-based static part is employed to achieve the tunable EIT-like phenomenon^30,52^, which requires a substrate and thus induces the adverse effect mentioned above. In this work, the bar-pair component was arranged in a plane without a substrate and then successfully fabricated through advanced MEMS fabrication technology. In other words, a high absolute transmittance is obtainable in this self-supporting metamaterial. This is the first time that this type of MEMS metamaterial has been numerically and experimentally demonstrated to our knowledge. In addition, the simple bar-pair shape is used to provide tractability in device fabrication; therefore, a high preparation accuracy of the structure can be feasible, which is significant for generating a strong EIT phenomenon. Moreover, the device is readily miniaturized and integrated into a real optical system by taking advantage of MEMS technology. This filter offers dynamic control of the transmittance and phase of THz waves, which may greatly promote the development of THz-related practical applications such as THz medical or biological detection and THz communications.

## Results

**Figure 1:**
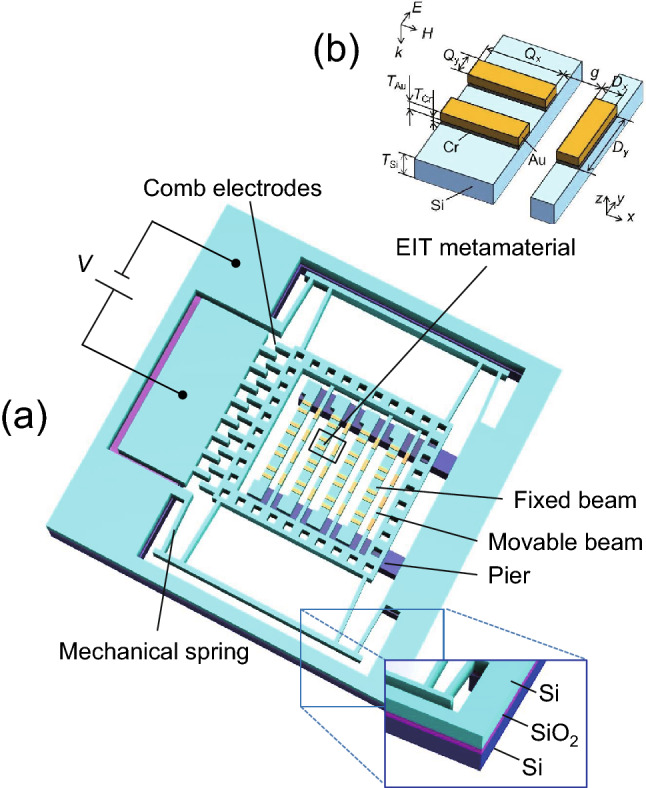
(**a**) Schematic view of the proposed MEMS integrated device. The close-up view (lower right) shows the overlapping three layers of the SOI wafer. (**b**) Unit cell of the EIT metamaterial, consisting of a cut wire and a wire pair with the following geometrical parameters: Q_x_ = 42.5 µm, Q_y_ = 10 µm, D_x_ = 10 µm, D_y_ = 48.5 µm, T_Si_ = 3.5 µm, T_Cr_ = 5 nm, and T_Au_ = 200 nm. The periods of each unit in both the x and y directions are 120 µm.

A schematic of the proposed MEMS integrated EIT filter is shown in Fig. 1a, which mainly consists of a gold EIT metamaterial and the mechanical drive system, including high-resistivity silicon supporting bridges and a comb-drive actuator. Each unit cell of the EIT metamaterial (Fig. 1b) is composed of a wire pair fixed on the silicon supporting bridges and a bar stacked on a movable beam that can be displaced by the comb-drive actuator through the electrostatic force. Electrostatic regulation was chosen due to its low power consumption and high tunability^24^. The distance between the bar and the wire pair, denoted g, is initially 30.8 µm and is mechanically changeable. When a y-polarized THz wave is normally incident on the gold metamaterial surface, the bar serves as a dipole resonator, directly coupling with the external light, which corresponds to the bright mode. The wire pair works as a quadrupole resonator with weak interaction with the incident waves, namely, the dark mode. The coupling between these two excitation modes, regarding the EIT analog, is controlled by adjusting the distance between the two resonators, i.e., controlling the lateral displacement of the bar through the mechanical actuator.

**Figure 2:**
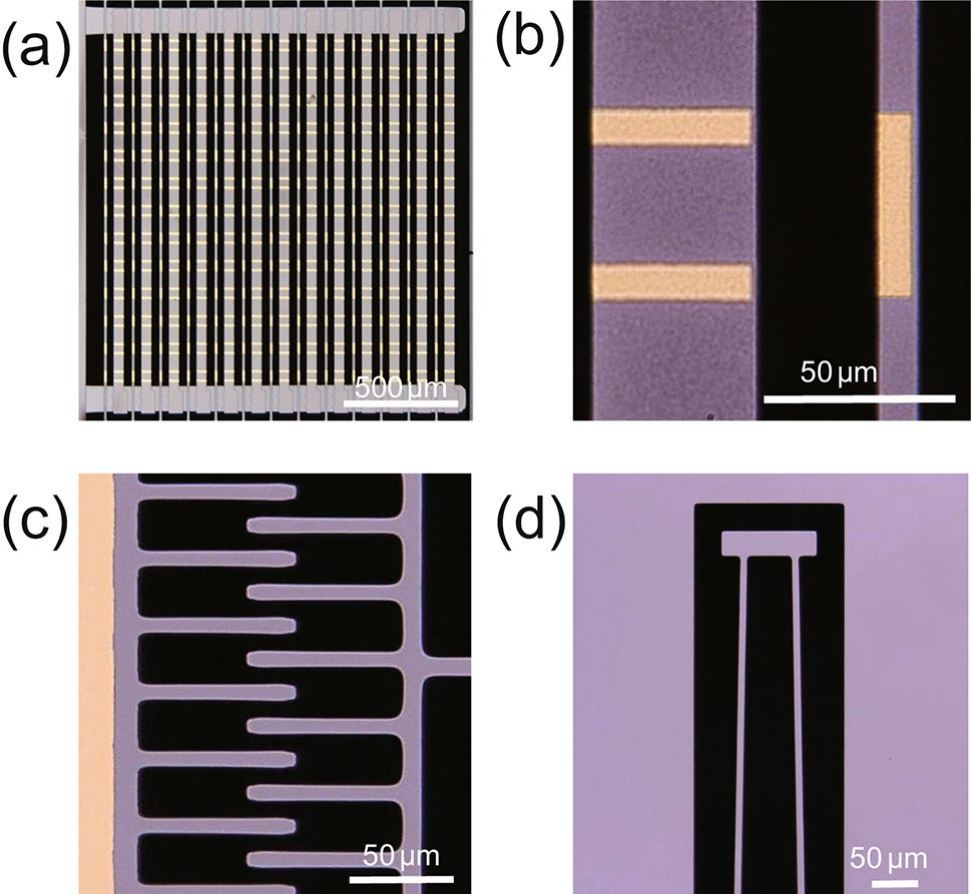
(**a**) Overview and (**b**) unit cell of the EIT metamaterial of the fabricated device. Magnified images of the (**c**) comb drive and (**d**) mechanical spring of the actuator.

**Figure 3:**
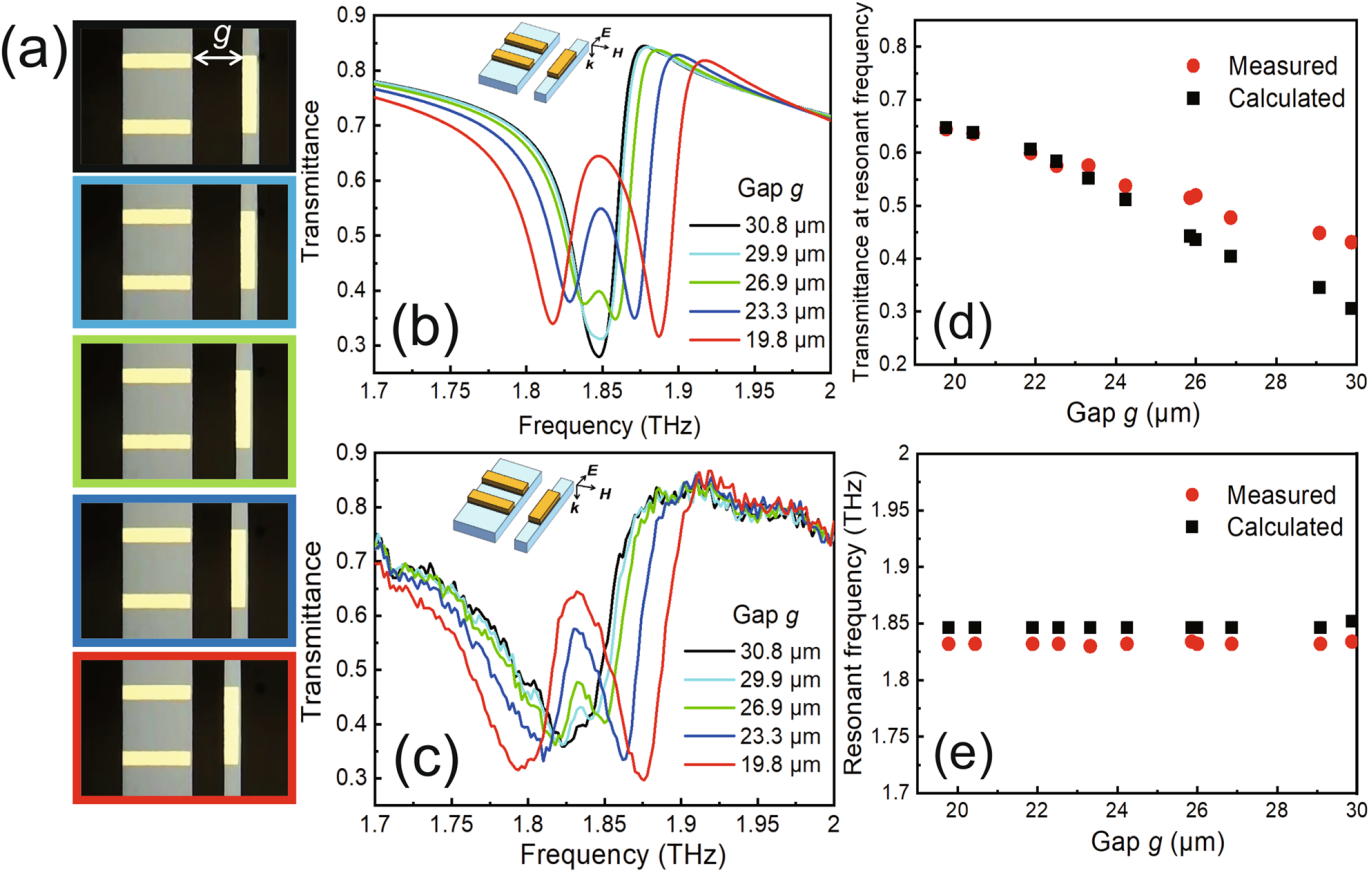
(**a**) Reconfigurable unit cell of the EIT metamaterial with a resonator gap from 30.8 to 19.8 µm. (**b**) Calculated and (**c**) measured EIT-like transmittance spectra of the device. Comparison of the (**d**) transmittance at the resonant frequency and (**e**) resonant frequency between the measured (red dots) and calculated (black squares) results. The images in (**a**) correspond to the spectra lines of the same color in (**b**) and (**c**). The insets in (**b**) and (**c**) show the polarization of the incident electric field.

An image of the fabricated device was captured by an optical microscope, as shown in Fig. 2 (the fabrication process is illustrated in the Methods section). The close-up views of the unit cell and the mechanical drive system reveal excellent structural sharpness and geometric accuracy. The dimensions of the fabricated EIT metamaterial are very close to the designed values, with a maximum manufacturing error of 0.6 µm, as shown in Table 1. The high fabrication accuracy enables the device to achieve strong EIT-like characteristics. A numerical simulation of the modulator was carried out using rigorous coupled-wave analysis (RCWA) based on Maxwell’s equations (details of the simulation conditions are shown in the Methods section). The calculated transmittance spectra of the device are plotted in Fig. 3. When the metamaterial is in the initial state with g equal to 30.8 µm (see the top image with a black frame in Fig. 3a), the transmittance spectrum of the device presents a deep dip at 1.846 THz with a minimum transmittance of 27.6%, as shown in Fig. 3b, which well matches the transmittance spectra of a single bar^16^. This result indicates that the gold bar acting as a dipole antenna is strongly excited by the external field, producing a broadband optical resonance in the transmittance spectrum, whereas the quadruple oscillator does not contribute to the excitation. In other words, the bright mode is dominant, and coupling between the two excitation modes does not occur in the case of a large g. A transmittance peak appears at the resonant frequency of 1.846 THz when g decreases to 26.9 µm, which means that the two excitation modes begin to couple due to the close proximity of the dipole and quadruple oscillators. The transmittance peak significantly strengthens and reaches a maximum of 64.7% as g decreases to 19.8 µm. The transmittance at the resonant frequency and the resonant frequency as a function of g are shown in Fig. 3d,e, respectively. A high transmittance modulation rate of 57.3% for THz waves around 1.846 THz is obtained by the device owing to the EIT-like effect in the metamaterial; here, the modulation rate is defined as ΔT/T_Max_, where T is the transmittance at the resonant frequency and ΔT is T_Maximum _− T_Minimum_. Note that almost no resonant frequency shift occurs.

**Table 1:** Comparison between the designed and fabricated values of the metamaterial dimensions.

	Q_x_ (µm)	Q_y_ (µm)	D_x_ (µm)	D_y_ (µm)
Designed value	42.5	10	10	48.5
Fabricated value	42.1	9.8	9.6	47.9

Next, the EIT-like phenomenon of the device is demonstrated by measuring the transmittance spectra via a THz optical measurement system (details of the characteristic system are shown in the Methods section) under various bias voltages. A DC power supply (Keithley Instruments, Model 2410) was used as the voltage source, the back surface of the substrate and the movable comb tooth beam were grounded, and a drive voltage was applied to the fixed supporting bridges. The applied drive voltages of 0, 100, 150, 200, and 250 V lead to distances g of 30.8, 29.9, 26.9, 23.3, and 19.8 µm, respectively. As shown in Fig. 3c, the classical dipole-yield resonance in the transmittance spectrum with a minimum transmittance of 39.5% is observed at 1.832 THz without any applied voltage. When the drive voltage increases to 150 V and g decreases to 26.9 µm via electrostatic force actuation, a transparency window clearly appears around the resonant frequency, namely, EIT-like behavior occurs. As g dynamically decreases to 19.8 µm as the drive voltage increases to 250 V, the modulation depth of the transparency window is controllably enhanced to 64.5%, in good agreement with the calculated results shown in Fig. 3d,e. The device successfully works as an actively tunable filter for waves around 1.832 THz based on the EIT-like behavior via simple adjustment of the applied voltage. As an example, a dynamic modulation demonstration of the fabricated metamaterial in which voltages of 0 and 200 V are periodically applied to the MEMS actuator to change g from 30.8 to 23.3 µm can be viewed in a Supplementary Video 1. A transmittance modulation rate of 38.8% for THz waves around 1.832 THz is experimentally achieved by applying the drive voltage. This performance makes the filter promising for THz optical switches, THz communications and other THz-wave-related applications.

**Figure 4:**
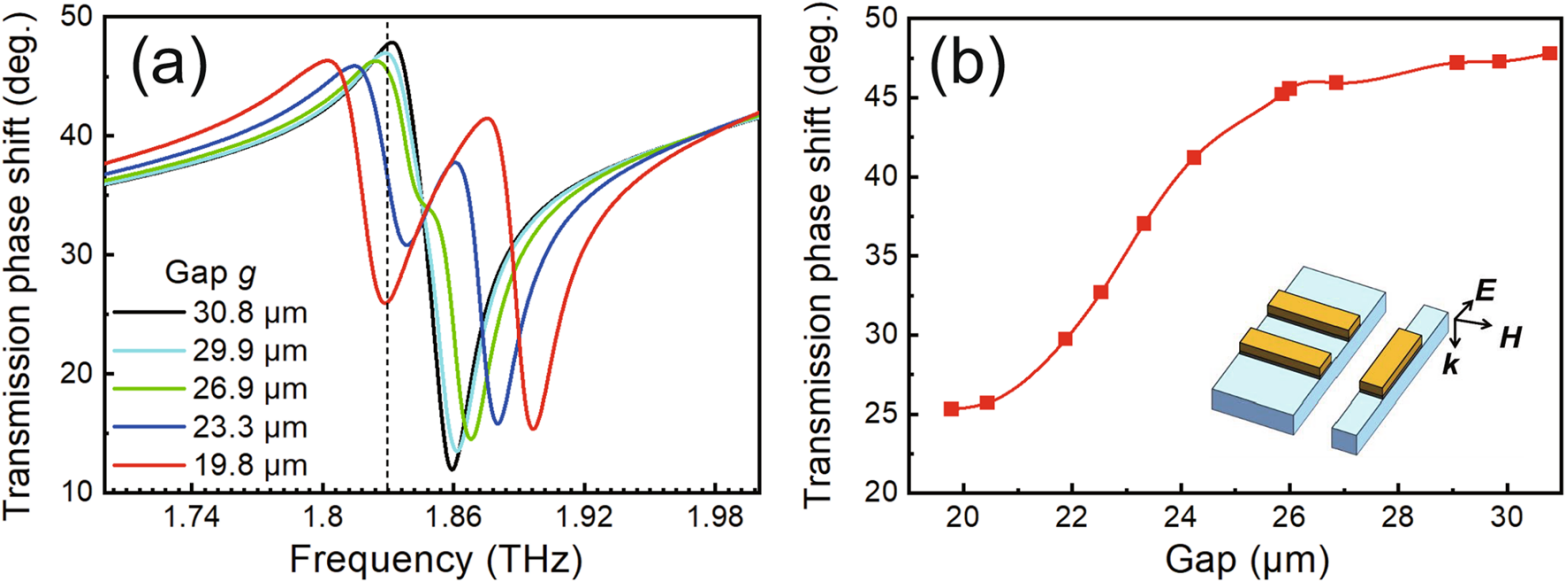
(**a**) Calculated transmission phase shift of the device with g ranging from 30.8 to 19.8 µm. The black dashed line shows the resonant frequency position. (**b**) Relationship between the resonator gap and the transmission phase shift at the resonant frequency.

Another well-known advantage of EIT analogs is their adjustable dispersion properties, which endow the filter with multifunctionality, such as phase shifting^53^, for THz waves. Therefore, the transmission phase spectra associated with the dispersion properties of the device were derived from the decline in the real and imaginary parts of the transmission coefficient based on the RCWA method, as plotted in Fig. 4a. The transmission phase shift varies with the g value of the unit cell. At the transparency window frequency of 1.832 THz, the phase shift as a function of the g value of the device is plotted in Fig. 4b. It reaches 47.8 degrees at a g of 30.8 µm and remains almost constant when g is greater than 26 µm; it then experiences a steep linear drop to 25.3 degrees when g decreases to 19.8 µm step by step, which is attributed to the occurrence of coupling between the two excitation modes, well matching the above transmittance results. The dispersion properties of the filter can be dynamically manipulated by MEMS actuation, which may benefit applications in active phase control.

**Figure 5:**
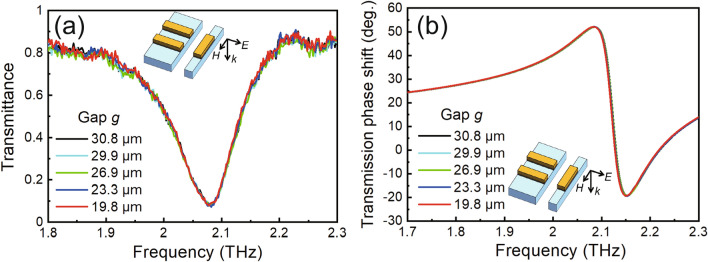
(**a**) Measured transmittance spectra and (**b**) transmission phase shift of the device under an incident electric field perpendicular to the cut wire.

The polarization properties of the device were also investigated by numerical calculations. As Fig. 5a shows, the typical spectral response produced by the dipole resonator instead of the quadruple resonator of the wire pair is observed under x-polarized incidence. The bar is unexcited because the external electric field is vertical to it. As g changes from 30.8 to 19.8 µm, EIT-like behavior does not occur due to the absence of coupling between the dipole and quadruple oscillators, i.e., the absence of an induced reduction in radiation losses. Likewise, the device loses the phase-shift behavior under x-polarized incidence (see Fig. 5b), which shows that this EIT metamaterial serves as a polarization-dependent filter for THz waves.

### Discussion

**Figure 6:**
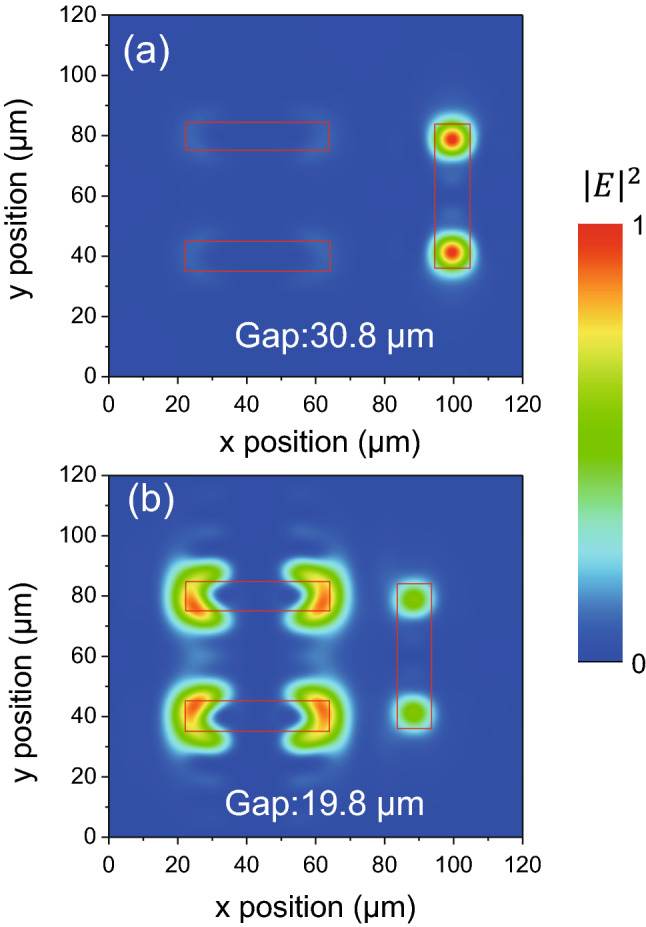
Normalized electric field intensity distributions of the EIT metamaterial unit with a resonator gap of (**a**) 30.8 µm and (**b**) 19.8 µm.

To further verify the nature of the EIT analog in the proposed device, the electric field intensity distribution on the surface of a unit cell under the on and off states of the EIT phenomenon was calculated and is presented in Fig. 6, which shows the results for a y-polarized wave at a resonant frequency of 1.846 THz. For the initial state of the device with g equal to 30.8 µm (Fig. 6a), the electric field intensity is concentrated on both ends of the bar, whereas almost none of it is distributed around the wire pair. This observation indicates that a strong bright mode of the bar is directly excited by the external electromagnetic field, contributing to the spectral resonance, and that the dark mode of the quadruple oscillator is completely uncoupled from the bright mode. When the bar and wire pair approach each other with g equal to 19.8 µm, the dark mode is greatly excited by the bright mode through near field coupling owing to the interference generated between the two modes. Therefore, the electromagnetic energy trapped in the dipole excited mode can be transferred to the quadruple excitation, resulting in localization of the electric field intensity at the ends of the wire pair, as shown in Fig. 6b. Moreover, the significant weakening of the electric field intensity at both ends of the bar reveals the dramatic suppression of the bright mode, namely, the significant suppression of the radiation losses, which leads to the EIT spectral characteristic occurring.

The clear EIT phenomenon reveals the coupling process between the two excitation pathways in the proposed metamaterial: the bright and dark modes excited by the dipole and quadruple oscillators work separately when a large distance exists between the bar and the wire pair, and the dipole antenna suffers very large losses due to the radiative damping caused by the plasmonic transformation and the nonradiative damping arising from the intrinsic metal loss^30^. When the bar moves close to the wire pair, interference between the two oscillators is generated, inducing a strong coupling of the bright and dark modes, which results in suppression of the radiative losses of the dipole oscillator, giving rise to a dramatically enhanced transmittance in a narrow wave range.

**Figure 7:**
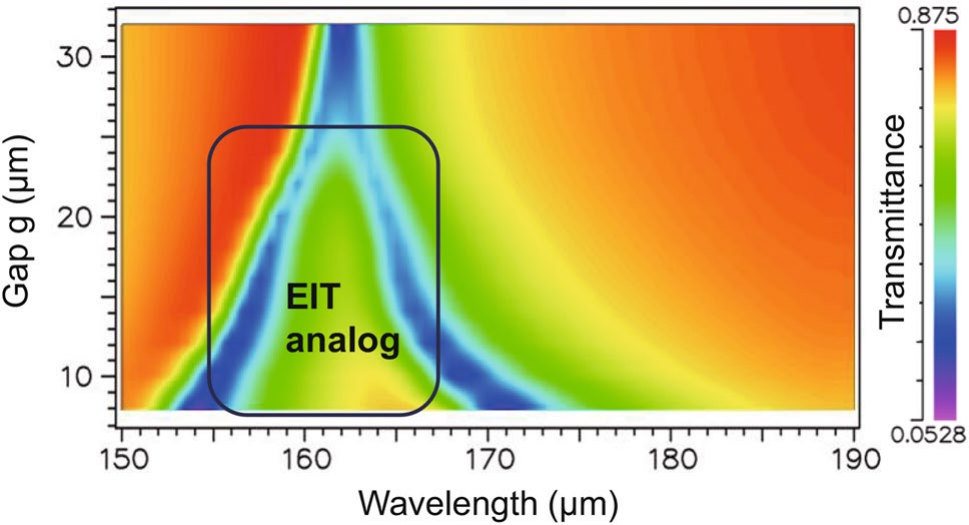
Calculated transmittance of the device as a function of the gap distance and the incident wavelength [drawn with SYNOPSYS DiffractMOD 2020.09–1 (https://www.synopsys.com/ja-jp/japan/products/rsoft-device.html)].

Notably, the central resonant frequency of the EIT-like spectra is fixed, whereas the frequencies of the low-frequency and high-frequency dips exhibit a redshift and a blueshift, respectively, which are observed in both the simulation and measurement results (Fig. 3b,c). Figure 7 shows the calculated transmittance of the device as a function of the gap distance and the incident wavelength. The transparent window (green triangular area within the frame) gradually broadens as the gap decreases, i.e., as the bright-dark mode coupling becomes stronger. In the MEMS metamaterial reported by Liu et al.^30^, a bar-pair component serving as the bright mode and dark mode was used; the EIT-like phenomenon generated in the infrared range had a fixed central resonant frequency, while the frequencies of the two dips shifted following the same trend as that in our work. In the metamaterial reported by Xu et al.^51^, the EIT-like phenomenon was observed in the THz range. The metamaterial consisted of a bar and an SRR pair, which were regarded as the bright and dark modes, respectively. By changing the distance between these two modes, the transmittance spectra showed the same dip-shift phenomenon. Unfortunately, the mechanism of the dip shifts in the EIT spectra is still unclear, which is an interesting phenomenon worthy of deep investigation in the near future.

In this work, to obtain high preparation accuracy and sharpness of the metamaterial in fabrication, a simple bar-pair shape with subtly designed geometrical parameters is employed. Moreover, by designing the shape of the metamaterial to achieve multi-coupling modes^51^, transparent multi-windows are possible in our system. Nevertheless, there are a few limitations of this work. Due to the high voltage (~ 250 V) requirements of the electrostatic actuator, problems of complex electronic circuits to provide the high voltage and material compatibility arise, which should be improved in the next step.

## Conclusions

In summary, an actively tunable THz filter based on an EIT metamaterial hybridized with MEMS was numerically and experimentally proposed. The metamaterial, with a self-supporting structure, consists of a gold bar and a wire pair without a substrate, which could work without the extra background intensity and interference waveforms that arise from substrates. For y-polarized waves around 1.832 THz, the transmittance could be dynamically manipulated to a maximum of 64.5% with a modulation rate of 38.8% by controlling the EIT behavior of the proposed filter, namely, by changing the distance between the bar and wire pair, which was successfully demonstrated by applying different drive voltages from 0 to 250 V. In addition, the filter can actively regulate the transmission phase for 1.832 THz waves between 47.8 degrees and 25.3 degrees owing to the tunable dispersive properties. Since the device is readily miniaturized and integrated into a real optical system by taking advantage of MEMS technology, the polarization-dependent filter is significantly favorable for active modulation, switching, memory and other practical applications in the THz wave band.

## Method

### Numerical calculation

The numerical calculation was implemented via RCWA, which enables calculation of the exact solutions for periodic structures based on Maxwell’s equations. The software SYNOPSYS Diffract MOD with the version number of 2020.09-1 was used to obtain the simulation results. A plane wave light source with an electric field polarized along the bar long axis was used. In both the x and y directions of the structure, periodic boundary conditions were set. The order of the Fourier series expansion considered in the simulation was set from -10 to + 10 in both the x and y directions. The complex refractive indices of Au and Cr, used as the materials of the metamaterial in the model, were given by D. Rakić et al*.*^54^.

### Fabrication

**Figure 8:**
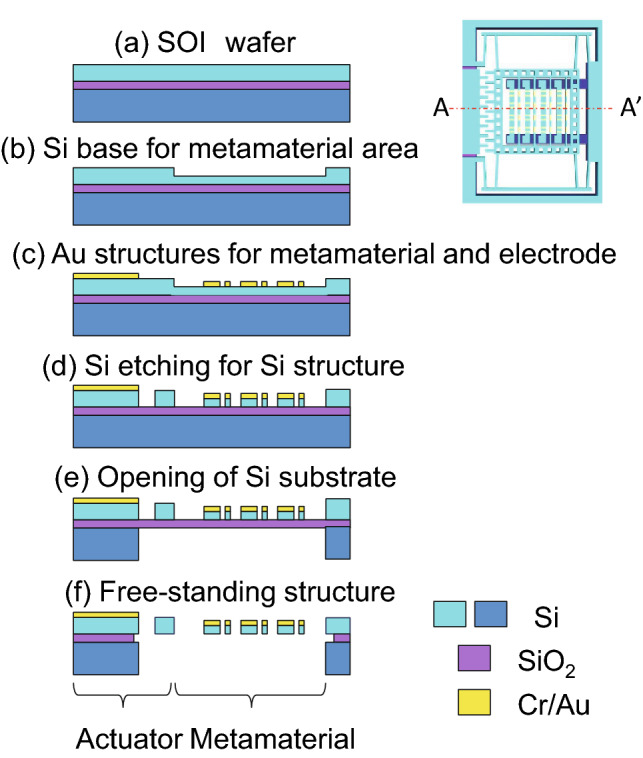
Fabrication processes of the MEMS-driven EIT device illustrated in the cross-section profile along A-A’.

The fabrication of the EIT metamaterial integrated with MEMS is illustrated in Fig. 8. A cleaned silicon-on-insulator wafer was used (Fig. 8a). First, the top silicon within the metamaterial array area was thinned to a thickness of 3.5 μm through photolithography (PMER P-LA900PM photoresist) and inductively coupled plasma-reactive ion etching (ICP-RIE) (Fig. 8b). Second, Cr/Au films with thicknesses of 5/200 nm were deposited on the top Si layer by a sputtering machine, and electrodes and a metamaterial array structure were formed by the ICP-RIE process with a shaped photoresist (PMER P-LA900PM) as a mask (Fig. 8c). Next, photolithography and etching were carried out on the top Si layer to form the comb drive and spring structures for the actuator (Fig. 8d), followed by etching of the backside silicon within the movable area by using a negative resist (MicroChem Co., SU-8 2100) as a mask and ICP-RIE (Fig. 8e). Finally, the SiO_2_ underneath the etched Si part was sacrificially removed by hydrofluoric acid vapor (SPTS Technologies Ltd., uEtch) to release the actuator and obtain the free-standing structures of the proposed EIT device (Fig. 8f).

### Characterization

**Figure 9:**
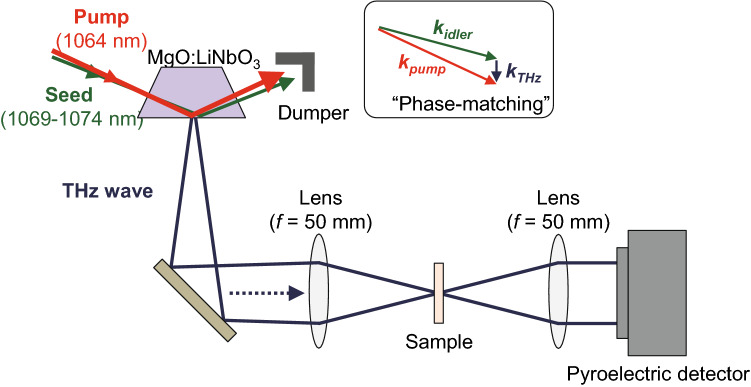
Schematic of the optical measurement system.

Figure 9 schematically depicts the utilized optical measurement system^55^. The optical system was set to satisfy the phase matching condition for the pumped light, the injection light, and the terahertz wave (the inset of Fig. 9). MgO:LiNbO_3_ was used for the nonlinear optical crystal. The collimated THz emissions were collected by a lens with a focal length of 50 mm and directed onto the sample, and then, the THz rays that penetrated the sample, carrying information from the sample, were gathered by another lens with a focal length of 50 mm and output to a pyroelectric detector. The spot diameter of the focused THz wave was approximately 1.5 mm. The transmittance was averaged by taking four measurements at one point from 1 to 2.5 THz at an interval of 2 GHz, which was carried out under a nitrogen atmosphere to avoid the absorption peak of water. All the measured transmittance spectra plotted in this article were the absolute transmittance spectra obtained from the measurement system, which did not undergo normalization. A DC power supply (Keithley Instruments, Model 2410) was employed to realize an adjustable voltage for generating the electrostatic force in the comb drive, which regulates the displacement of the gap in each unit. A maximum displacement of 11 μm, from an initial gap of 30.8 to 19.8 μm, was achieved by applying a voltage of 250 V.

## Supplementary information

Supplementary Legends.

Supplementary Video 1.
