# Supplementary material for: Actively tunable THz filter based on an electromagnetically induced transparency analog hybridized with a MEMS metamaterial
Source: Sci Rep. 2020 Nov 30;10:20807. doi: 10.1038/s41598-020-77922-1 (PMC7705675; doi:10.1038/s41598-020-77922-1)
Supplement: Supplementary file 1 — Supplementary Legends. [file 41598_2020_77922_MOESM1_ESM.docx]

**Actively tunable THz filter based on an electromagnetically induced transparency analog hybridized with a MEMS metamaterial**

Ying Huang^1^, Kenta Nakamura^2^, Yuma Takida^3^, Hiroaki Minamide^3^, Kazuhiro Hane^2^, Yoshiaki Kanamori^1^*

1. Department of Robotics, Tohoku University, Sendai 980-8579, Japan
2. Department of Finemechanics, Tohoku University, Sendai 980-8579, Japan
3. RIKEN Center for Advanced Photonics, RIKEN, Sendai 980-0845, Japan

**kanamori@meta.mech.tohoku.ac.jp*

**Supplementary video information**

This supplementary video demonstrates a dynamic modulation process of the fabricated metamaterial. When 0 and 200 V are periodically applied to the MEMS actuator, the distance between the bar and wire pair of the metamaterial dynamically switches between 30.8 and 23.3 µm, thus modulating the EIT behavior. A DC power supply (Keithley Instruments, Model 2410) was used as the voltage source.

The working state of the EIT metamaterial is shown, taking this video as an instance.
